# Sleep Problems in Children with ADHD: Associations with Internalizing Symptoms and Physical Activity

**DOI:** 10.1007/s10803-024-06623-9

**Published:** 2024-11-01

**Authors:** Xiao Liang, Mengping Zhao, Li Su, Justin A. Haegele, Richard H. Xu, Jiayue Li, Jinxian Guo, Andy Choi-Yeung Tse, Shirley X. Li, David H. K. Shum

**Affiliations:** 1 Department of Rehabilitation Sciences, The Hong Kong Polytechnic University, Hung Hom, Kowloon, Hong Kong SAR, China; 2 The Research Institute for Sports Science and Technology, The Hong Kong Polytechnic University, Hong Kong SAR, China; 3 School of Public Health, Lanzhou University, Lanzhou, China; 4 Department of Human Movement Sciences, Old Dominion University, Norfolk, VA USA; 5 Centre for Early Child Development, Gansu Maternity and Child Health Care Hospital, Lanzhou, China; 6 Department of Health and Physical Education, The Education University of Hong Kong, Hong Kong SAR, China; 7 Department of Psychology, University of Hong Kong, Hong Kong SAR, China; 8 The State Key Laboratory of Brain and Cognitive Sciences, The University of Hong Kong, Hong Kong SAR, China

**Keywords:** ADHD, Sleep, Physical activity, Internalizing symptoms, Children

## Abstract

Children with attention deficit hyperactivity disorder (ADHD) experience high rates of sleep problems and are at increased risk for developing internalizing problems. This study aimed to examine the association of sleep problems and physical activity with internalizing symptoms in children with ADHD. This cross-sectional study included 188 children with ADHD (M age = 8.60 ± 1.38, 78.7% boys). Self-reported questionnaires were used to assess sleep problems (Pittsburgh Sleep Quality Index [PSQI]) and internalizing symptoms (Depression Anxiety Stress Scale 21 [DASS 21]). The presence of sleep problems was defined as a PSQI score > 5. Physical activity was recorded by an ActiGraph GT9X Link accelerometer for 7 consecutive days. In total, 111 children with ADHD presented with sleep problems (59%). Compared with their counterparts without sleep problems, children with sleep problems spent less time in daily moderator-to-vigorous physical activity (MVPA) (F = 15.35, η2 = .079), had a lower proportion of meeting the WHO-recommended 60 min of daily MVPA guideline (F = 9.57, η2 = .050), and showed more internalizing symptoms: depression (F = 10.09, η2 = .053), anxiety (F = 15.84, η2 = .081), and stress (F = 6.98, η2 = .037). BMI, daytime dysfunction of PSQI, and MVPA guideline attainment were significantly associated with internalizing symptoms in children with ADHD. Daytime dysfunction of PSQI is associated with more severe internalizing symptoms, and MVPA guideline attainment may reduce the likelihood of developing depression and anxiety in children with ADHD. Future studies are needed to examine the long-term effects of sleep on internalizing symptoms and the effects of PA-based interventions on sleep and internalizing symptoms in children with ADHD, respectively.

## Introduction

Attention-Deficit/Hyperactivity Disorder (ADHD) is a pervasive childhood psychiatric disorder characterised by a persistent condition of age-inappropriate levels of inattention, impulsivity, and hyperactivity (American Psychiatric Association, 2013), affecting approximately 5–6% of school-aged children (Sayal et al., 2018). Individuals with ADHD may experience significant comorbidities beyond the core symptoms of ADHD, including those related to psychological difficulties, such as internalizing symptoms (i.e., depression, anxiety, and social withdrawal) and externalizing symptoms (i.e., aggression, conduct problems and being oppositional), sleep disturbances and physical inactivity (Gillberg, 2010). Together, all these symptoms can cause substantial deficits in the daily routine of these children (Becker, 2020).

Sleep is essential for the overall health of children (Matricciani et al., 2019), yet sleep problems are common in children with ADHD (Becker, 2020). Increasing evidence suggests that behavioural sleep problems are significantly more common in children with ADHD than in children without ADHD, with estimated prevalence rates ranging from 25 to 50% (Becker, 2020). The most common subjective sleep complaints in children with ADHD include sleep latency delay, bedtime resistance, nocturnal awakenings, and daytime sleepiness (Cortese et al., 2009). A recent meta-analysis concluded that compared with peers without ADHD, children and adolescents with ADHD show a longer sleep latency and decreased sleep efficiency, as measured by actigraphy (Liang et al., 2023a). Indeed, there is evidence to suggest that sleep problems may exacerbate existing ADHD symptoms, academic problems, and adverse effects on health-related quality of life for children with ADHD and their families (Hvolby, 2015; Langberg et al., 2013). Furthermore, a number of studies have demonstrated that behavioural sleep problems are related to a range of internalizing and externalizing symptoms in children with ADHD, resulting in reduced quality of life (QoL) and academic underachievement (Becker et al., 2019; Dimakos et al., 2021).

Despite the high prevalence of sleep problems, the evidence of how sleep problems may influence daytime functioning in children with ADHD is limited (Hansen et al., 2014). Recent reviews summarized that externalizing and internalizing problems contribute to dysfunction in youth with ADHD and can be amplified by behavioural sleep disturbances (Bondopadhyay et al., 2022; Dimakos et al., 2021). Specifically, there is a significantly positive association between reported sleep problems and both internalizing and externalizing in youth with ADHD ranging from preschool to adolescence (Dimakos et al., 2021). However, while the causal associations between sleep disturbances and externalizing symptoms (e.g., oppositional behaviours, inattention, and behavioural disorders) were consistently found in youth with ADHD, the relationships between sleep problems and internalizing problems (e.g., depression and anxiety) were equivocal (Dimakos et al., 2021). In addition, a previous study of 239 children with ADHD found that those with ADHD with moderate-to-severe sleep problems showed more severe ADHD symptoms than children with no or mild sleep problems (Sung et al., 2008). In another cross-sectional study of 63 children with ADHD, those with insomnia symptoms showed more severe ADHD symptoms and poorer cognitive function performance compared with children with ADHD without insomnia and healthy controls (Li et al., 2022). Despite these findings, very limited research has been developed to identify predictors of internalizing symptoms in children with ADHD, which is critical to designing tailored intervention programs (Becker et al., 2015).

While the impact of behavioural sleep problems on internalizing symptoms is emerging in youth with ADHD, the mechanisms associated with sleep problems in children with ADHD appear multi-factorial, including biological (e.g., genetic factors, circadian rhythm alterations), neurological (overlap in disrupted brain circuitry), psychological (conduct problems) (Dong et al., 2024), and environmental (e.g., night media use) (Becker, 2020; Bondopadhyay et al., 2022; Lycett et al., 2015). Given the risks posed by internalizing symptoms and the fact that behavioural sleep disturbances can lead to an increased risk of internalizing symptoms in children with ADHD (Dimakos et al., 2021), sleep problems have emerged as a pivotal risk factor for internalizing symptoms in youth with ADHD. However, there is still a poor understanding of the behavioural mechanisms by which sleep problems exert effects on internalizing symptoms in children with ADHD.

PA has consistently been reported to have beneficial effects on improving sleep problems (e.g., decreased sleep latency) (Liang et al., 2022a, 2022b), as well as reducing depression (Gawrilow et al., 2016; Liang et al., 2023b) and stress (Liang et al., 2023b) in children with ADHD. Sleep problems and physical inactivity are significantly more common in children with ADHD (Becker, 2020). Growing evidence shows an association between ADHD and decreased PA, with subjective reports indicating that children with ADHD tend not to meet the World Health Organization’s (WHO’s) minimum guidelines of 60 min per day of moderate-to-vigorous PA (MVPA) (Tandon et al., 2019). For example, one recent study found that MVPA guideline attainment and sleep duration significantly predicted locomotor skills development in children with ADHD. Moreover, significantly lower accelerometer-measured MVPA was observed in children with ADHD compared to those without ADHD (Liang et al., 2023c). In addition, one recent pilot study reported beneficial training effects of a 12-week PA intervention on psychological resilience and internalizing symptoms (e.g., anxiety/depressed, withdrawn/depressed, and somatic complaints) and externalizing symptoms (e.g., rule-breaking behaviour and aggressive behaviour) in children with ADHD (Liang et al., 2024). However, few studies have considered how PA may affect the associations between sleep and internalizing symptoms in children with ADHD.

Therefore, the current study aimed to (a) compare internalizing symptoms and PA between children with ADHD and sleep problems with children with ADHD but without sleep problems; and (b) determine the association among sleep problems, PA and internalizing symptoms among children with ADHD. Overall, we hypothesized that (a) children with ADHD and behavioural sleep problems would have more severe internalizing symptoms and lower PA levels as compared with children with ADHD but without behavioural sleep problems; (b) there would be a significant association of sleep problems and physical activity with internalizing symptoms.

## Methods

### Study Design and Participants

This cross-sectional study recruited 188 children with ADHD (M age = 8.60 ± 1.38, 78.7% boys) (with or without behavioural sleep disturbances) aged between 6 and 12, who were outpatients attending a children’s hospital in Lanzhou, China. The clinical diagnosis of ADHD (any subtypes) was confirmed by expert child psychologists based on the Diagnostic and Statistical Manual of Mental Disorders-Fifth version (DSM-5) (American Psychiatric Association, 2022). All the recruited children met the following study criteria: (1) IQ of at least 75 on Wechsler Intelligence Scale for Children, 4th Edition (WISC-IV) (Zhang, 2009) as assessed by a clinical psychologist; (2) absence of prominent medical conditions that limited PA capability (e.g., asthma, cardiac disease) was also checked by pediatricians; (3) absence of neuropsychiatric disorders (e.g., bipolar disorder, autism spectrum disorder, substance abuse) (American Psychiatric Association, 2022). All recruitment and participant screening was carried out by pediatricians following the standard protocol at the hospital.

### Procedure

This study’s design followed the Declaration of Helsinki’s ethical standards and was approved by the Institutional Review Board for Human Subjects (Clinical), The Hong Kong Polytechnic University (Reference No: HSEARS20230314005). Informed consent was obtained from the participants’ parents or guardians. Participants individually visited the consulting room with their parents/guardians. Weight and height were measured by clinicians, and body mass index (BMI) was calculated by dividing body mass (kg) by height squared (m^2^). All participants individually completed the two questionnaires (i.e., PSQI and DASS 21) with the assistance of trained research assistants. Then, each participant was asked to wear an Actigraphy on the non-dominant wrist, and a logbook was also provided for his/her parents to record put-on/take-off time and reasons. After seven days of wearing it, the participant’s parents/guardians brought or mailed the Actigraphy back to the hospital.

### Measures

#### Behavioural Sleep Disturbances

Sleep disturbances were measured by the self-reported Chinese version of the Pittsburgh Sleep Quality Index (PSQI) questionnaire (Liang et al., 2022a, 2022b). This measure consists of 18 questions and forms seven-component scores ranging from 0 to 3 (i.e., sleep quality, sleep latency, sleep duration, habitual sleep efficiency, sleep disturbances, sleep medication and daytime dysfunction). The sum of each component is the total score, which ranges between 0 and 21, where a higher score describes poorer sleep quality. A total PSQI score greater than 5 has been validated as being highly sensitive and specific in distinguishing good from poor sleepers in children and adolescents with ADHD (Liang et al., 2023a). The cut-off score of 5 was used to determine the differences between children with ADHD and behavioural sleep disturbances and children with ADHD but without sleep disturbances. Specifically, an overall score of 5 or higher indicates ADHD children with behavioural sleep disturbances, while an overall score of less than 5 indicates ADHD children without sleep disturbances (Zitser et al., 2022). The internal consistency was acceptable in the present study (α = 0.784).

#### Internalizing Symptoms

Internalizing symptoms were measured using the Chinese version of the Depression Anxiety Stress Scale 21 (DASS 21) (K. Wang et al., 2016). The DASS 21 is comprised of 21 items and designed to assess three dimensions of a 1-week state of negative affect (7 items in each of the depression, anxiety, and stress subscales). DASS 21 is rated using a four-point Likert scale from (0) never to (3) almost always. Higher scores indicate a more severe level of depression, anxiety, or stress. The Chinese version of the DASS 21 has been applied to Chinese children with ADHD and has been shown to be useful for this particular population (Liang et al., 2023b). The internal consistency of each subscale was reported to be acceptable in this study (depression: α = 0.77; anxiety: α = 0.73; stress: α = 0.75).

#### Physical Activity

Physical activity data used for analysis were time spent in MVPA, which was assessed by ActiGraph GT9X Link (ActiGraph, Pensacola, Florida, USA). All participants were asked to wear the actigraph around their non-dominant wrist for seven consecutive days (five weekdays and two weekend days) and were instructed to follow their typical daily routines and only take off the actigraph when they were taking baths or swimming. Participants were excluded if they had less than four valid days of wearing time (a minimum of three weekdays and one weekend day). MVPA values were calculated by averaging MVPA metrics for each participant's valid days. The Actigraph has been used extensively to measure physical activity and is well-validated in MVPA records in children with ADHD (Liang et al., 2023b, 2023c). Engaging an average MVPA for ≥ 60 min/day in valid days was considered to meet the WHO-recommended MVPA guideline (Liang et al., 2023c).

### Data Analysis

All statistical analyses were performed using the Statistical Package for the Social Sciences (SPSS, version 29.0). Participants were divided into two groups: poor sleepers (PS) (PSQI ≥ 5) and good sleepers (GS) (PSQI < 5). Independent t-tests or chi-square tests were used to compare the differences in background information (e.g., age, sex, IQ, height, weight, BMI, ADHD subtype, comorbidity, and medication) between the two groups. Then, a multivariate analysis of covariance controlling for age, sex, IQ, BMI, comorbidity, medication, and ADHD subtypes was carried out to compare the group differences in internalizing symptoms, physical activity and sleep characteristics. Bivariate correlations were calculated among our variables of interest: PSQI (i.e., sleep quality, sleep latency, sleep duration, habitual sleep efficiency, sleep disturbances, sleep medication and daytime dysfunction), MVPA, MVPA guideline attainment, and internalizing symptoms (i.e., depression, anxiety, and stress). Lastly, hierarchical regression models were used to predict internalizing symptoms (i.e., depression, anxiety, and stress). For all analyses, statistical significance was set at *p* < 0.05.

## Results

### Characteristics of the Participants

Table 1 shows the characteristics of the participants. Of the initially recruited 191 children with ADHD, 3 participants dropped out of the study due to their poor compliance with wearing the actigraphy and were excluded from data analyses, resulting in a final dataset of 188 children with ADHD. All participants had valid accelerometer data for at least four days, including three weekdays and one weekend day. Among the 188 ADHD participants in this study, 148 (78.7%) were boys, while 40 (21.3%) were girls. The average age of the participants was 8.6 ± 1.38 years old. The mean IQ of the participants was 92.88 ± 10.92. Regarding the ADHD subtype, the majority of the participants were diagnosed with ADHD Inattentive subtype (ADHD-I) (n = 105, 55.9%), followed by ADHD Combined subtype (ADHD-C) (n = 61, 32.4%) and the ADHD Hyperactive-Impulsive subtype (ADHD-H) (n = 22, 11.7%). In addition, 28.2% of the participants were diagnosed as having comorbid oppositional defiant disorder (ODD). Additionally, 10.6% of the participants reported regular use of medication for ADHD management. Based on a PSQI cutoff score of 5, the Good Sleeper (GS) group consisted of 77 ADHD children (41%) (M_age_ = 8.74, 80.5% boys) and the Poor Sleeper (PS) group consisted of 111 ADHD children (59%) (M_age_ = 8.51, 77.5% boys). There were no significant differences in age, sex, IQ, BMI, ADHD subtype, comorbidity, and medication between the good and poor sleep groups.

**Table 1:** Descriptive statistics in ADHD groups (n = 188)

Variable	Total (n = 188)	Good sleeper (n = 77)	Poor sleeper (n = 111)	*p*
Sex (n, % male)	148, 78.7%	62, 80.5%	86, 77.5%	.616
Age (years)	8.60 ± 1.38	8.74 ± 1.44	8.51 ± 1.34	.247
IQ	92.88 ± 10.92	91.64 ± 11.62	93.74 ± 10.37	.195
Weight (kg)	30.39 ± 10.39	30.81 ± 10.18	30.09 ± 10.57	.639
Height (cm)	132.38 ± 10.18	133.36 ± 9.86	131.86 ± 10.17	.316
BMI (kg/m^2^)	16.93 ± 3.41	16.97 ± 3.42	16.90 ± 3.42	.890
ADHD subtype (n, %)				
Inattention	105, 55.9%	43, 55.8%	62, 55.9%	.131
Hyperactivity	22, 11.7%	13, 16.9%	9, 8.1%	
Combined	61, 32.4%	21, 27.3%	40, 36.0%	
Comorbidity (n,%)				
Oppositional defiant disorder	53, 28.2%	25, 32.5%	28, 25.2%	.438
Tic disorder	7, 3.7%	2, 2.6%	5, 4.5%	
Anxiety	8, 4.3%	4, 5.2%	4, 3.6%	
Obsessive–compulsive disorder	3, 1.6%	0, 0%	3, 2.7%	
Medication (n,%)	20 (10.6%)	11, 14.3%	9, 8.1%	.177
Concerta	6	3	3	
Strattera	10	6	4	
Xiao’er Zhili Syrup (Kuihua)	3	2	1	
Jingling Oral Liquid	1	–	1	

*IQ* intelligence quotient, *BMI* body mass index

### Comparisons of Sleep Characteristics, Internalizing Symptoms and Physical Activity Level Between Good Sleepers and Poor Sleepers

Table 2 presents the GS/PS group comparison of all outcome variables. Compared to the GS group, the PS spent significantly less time in MVPA (F = 15.35, *η*^*2*^ = 0.079, *p* < 0.001) and had a lower proportion of meeting the MVPA guidelines (72.7% Vs 55.9%, F = 9.57, *η*^*2*^ = 0.050, *p* = 0.002). The PS group had significantly higher scores in depression (F = 10.09, *η*^*2*^ = 0.053, *p* = 0.002), anxiety (F = 15.84, *η*^*2*^ = 0.081, *p* < 0.001) and stress (F = 6.98, *η*^*2*^ = 0.037, *p* = 0.009), indicating more severe internalizing symptoms, compared to those without sleep disturbances. There were significant group differences in PSQI general scores (F = 257.98,* η*^*2*^ = 0.589, *p* < 0.001). Specifically, the PS group reported lower general sleep quality (F = 72.69, *η*^*2*^ = 0.288, *p* < 0.001), longer sleep latency (F = 57.50, *η*^*2*^ = 0.242, *p* < 0.001), less sleep duration (F = 8.69, *η*^*2*^ = 0.046, *p* = 0.004), lower sleep efficiency (F = 9.81, *η*^*2*^ = 0.052, *p* = 0.002), more sleep disturbances (F = 4.27, *η*^*2*^ = 0.023, *p* = 0.040), having more sleep medicine (F = 17.67, *η*^*2*^ = 0.089, *p* < 0.001), and more severe daytime dysfunction (F = 81.86, *η*^*2*^ = 0.313, *p* < 0.001) than in the GS group. In addition, the PS group had significantly later bedtimes than the GS group (F = 6.36, *η*^*2*^ = 0.034, *p* = 0.013).

**Table 2:** Comparisons of sleep characteristics, internalizing symptoms and physical activity level between good sleepers and poor sleepers

	Good sleeper (n = 77) M ± SD	Poor sleeper (n = 111) M ± SD	*F*	*p*	*η2*
Physical activity levels					
MVPA (mins)	78.11 ± 33.12	66.70 ± 25.89	15.35	< .001	.079
MVPA guideline attainment (yes/no), %	56/21, 72.7%	62/49, 55.9%	9.57	.002	.050
Internalizing symptoms (DASS-21)					
Depression	3.95 ± 3.13	5.47 ± 3.27	10.09	.002	.053
Anxiety	3.44 ± 3.06	5.34 ± 3.75	15.84	< .001	.081
Stress	5.26 ± 3.58	6.83 ± 3.84	6.98	.009	.037
Pittsburgh sleep quality index (PSQI)					
PSQI general score	2.84 ± 1.04	7.66 ± 2.38	257.98	< .001	.589
*Components*					
1—Sleep quality	0.38 ± 0.63	1.48 ± 1.01	72.69	< .001	.288
2—Sleep latency	0.58 ± 0.69	1.53 ± 0.85	57.50	< .001	.242
3—Sleep duration	0.27 ± 0.60	0.69 ± 0.79	8.69	.004	.046
4—Sleep efficiency	0.30 ± 0.65	0.77 ± 0.87	9.81	.002	.052
5—Sleep disturbances	0.92 ± 0.60	1.17 ± 0.77	4.27	.040	.023
6—Sleep medicine	0.08 ± 0.31	0.35 ± 0.71	17.67	< .001	.089
7—Daytime dysfunction	0.60 ± 0.71	1.68 ± 1.03	81.86	< .001	.313
Sleep–wake Pattern (PSQI)					
Bedtime (hh:mm)	22:02	22:20	6.36	.013	.034
Rise time (hh:mm)	07:11	07:15	0.27	.603	.002

PSQI cutoff of < 5 indicating “good sleeper”; PSQI cutoff of 5–21 indicating “poor sleeper”

*MVPA* moderate-to-vigorous physical activity, *BMI* body mass index, *M* mean, *SD* standard deviation, *P* P‐value, *N/A* not available; group comparison results controlling age, sex, BMI, IQ, comorbidity, medication ADHD subtype

### Association of Sleep Problems and Physical Activity (IV) with Internalizing Symptoms (DV)

Table 3 presents the correlations among sleep problems (PSQI general scores), internalizing symptoms (e.g., depression, anxiety, and stress), MVPA, and MVPA guideline attainment. The results of skewness (|skewness|< 3) and kurtosis (|kurtosis|< 10) tests showed that data were normally distributed (Kline, 2015). Sleep disturbance was positively related to anxiety and stress; daytime dysfunction was positively correlated to internalizing symptoms (depression, anxiety and stress) while negatively correlated to MVPA. MVPA guideline attainment was negatively associated with depression and anxiety.

**Table 3:** Association of sleep problems and physical activity (IV) with internalizing symptoms (DV) (r-values)

	1	2	3	4	5	6	7	8	9	10	12	13
1. Sleep quality^a^	–											
2. Sleep latency^a^	.417**	–										
3—Sleep duration^a^	.101	.331**	–									
4—Sleep efficiency^a^	.096	.352**	.749**	–								
5—Sleep disturbance^a^	.179*	.294**	.224**	.187*	–							
6—Sleep medicine^a^	.033	– .083	– .172*	– .184*	– .352**	–						
7—Daytime dysfunction^a^	.271**	.137	– .080	– .088	– .043	.481**	–					
8. Depression^b^	.231**	.170*	.147*	.090	.143	.016	.294**	–				
9. Anxiety^b^	.196**	.165*	.053	.056	.252**	– .056	.316**	.563**	–			
10. Stress^b^	.139	.119	.047	.055	.207**	– .008	.254**	.506**	.592**	–		
12. MVPA	– .136	– .012	.087	.160	.005	– .167*	– .183*	– .171*	– .137	.032	–	
13. MVPA guideline attainment	– .120	– .023	– .043	– .011	– .049	– .134	– .080	– .254**	– .195**	– .055	.719**	–
*Mean*	1.03	1.14	.52	.57	1.07	.24	1.23	4.85	4.56	6.19	71.38	–
*SD*	1.02	.92	.74	.82	.72	.60	1.05	3.29	3.60	3.81	29.52	–

Moderate-to-vigorous physical activity. For MVPA guideline attainment, 0 = without MVPA guideline attainment, 1 = with MVPA guideline attainment

^a^ Scores from the Pittsburgh Sleep Quality Index (PSQI)

^b^ Scores from the Depression Anxiety Stress Scale 21 (DASS 21)

^*^p < .05. **p < .01

N = 188

### Association of Sleep Problems and Physical Activity with Depression, Anxiety and Stress

A three-step hierarchical regression analysis was carried out to identify significant factors associated with internalizing symptoms such as depression, anxiety, and stress. The independent variables were entered as follows: Step 1 included covariates such as IQ, age, BMI, sex, ADHD subtype, comorbidity and medication use; Step 2 included the behavioural sleep problems variable such as sleep quality, sleep latency, sleep duration, sleep efficiency, sleep disturbances, sleep medication and daytime dysfunction, and Step 3 included the PA variables, including daily MVPA and MVPA guideline attainment. The results of the regression analysis related to the variables associated with internalizing symptoms are depicted in Table 4.

**Table 4:** Association of sleep problems and physical activity with depression, anxiety and stress

	Depression			Anxiety			Stress		
Predictor Variables	Model 1	Model 2	Model 3	Model 1	Model 2	Model 3	Model 1	Model 2	Model 3
*Step 1: Demographic*									
IQ	− 0.130	− 0.167*	− 0.133	− 0.097	− 0.128	− 0.103	− 0.086	− 0.109	− 0.108
Age	− 0.158*	− 0.138	− 0.114	− 0.023	− 0.077	0.010	− 0.127	− 0.116	− 0.101
BMI	0.073	0.058	0.055	0.178*	0.152*	0.150*	0.202*	0.178*	− 0185*
Sex	− 0.052	− 0.071	− 0.049	− 0.062	− 0.061	− 0.045	− 0.082	− 0.084	− 0.052
ADHD-HI subtype	− 0.128	− 0.061	− 0.085	− 0.055	− 0.011	− 0.027	0.064	0.096	0.061
ADHD-C subtype	0.021	0.030	0.066	− 0.025	− 0.007	0.019	0.109	0.120	0.123
Comorbidity	0.097	0.081	0.088	− 0.026	− 0.076	− 0.072	− 0.020	− 0.061	− 0.044
Medication use	− 0.025	− 0.018	− 0.002	− 0.098	− 0.065	− 0.053	− 0.022	0.005	0.012
*Step 2: Sleep*									
Sleep quality		0.154	0.112		0.072	0.041		0.060	0.049
Sleep latency		0.003	0.024		0.024	0.039		− 0.012	0.001
Sleep duration		0.166	.148		− 0.020	− 0.034		− 0.015	− 0.017
Sleep efficiency		− 0.048	− 0.071		0.045	0.028		0.046	0.013
Sleep disturbances		0.067	0.040		0.181*	0.162*		0.166	0.156
Sleep medicine		− 0.087	− 0.136		− 0.165	− 0.200		− 0.100	− 0.112
Daytime dysfunction		0.289**	0.319**		0.381**	0.042**		0.302**	0.325**
*Step 3: Physical activity*									
MVPA			0.125			0.087			0.176
MVPA guideline attainment			− 0.302**			− 0.218*			− 0.151
*R*^*2*^	0.068	0.211	0.257	0.063	0.252	0.276	0.066	0.186	0.197
*F*	1.620	3.058	3.456	1.506	3.858	3.817	1.569	2.614	2.454
*ΔR*^*2*^* (R*^*2*^* change)*	0.068	0.143	0.046	0.063	0.189	0.024	0.066	0.120	0.011
*ΔF (F change)*	1.620	4.451	5.297	1.506	6.195	2.877	1.569	3.625	1.204

Coefficients shown are standardized beta coefficients. MVPA, moderate-to-vigorous physical activity; *BMI* body mass index; *p < .05, **p < .01

For Sex, boy = 0, girl = 1. For ADHD subtype, ADHD-I = 0, ADHD-HI/ADHD-C = 1. For Comorbidity, no comorbid disorders = 0, comorbid disorders = 1

For medication use, medication-naïve = 0, medicated = 1

For MVPA guideline attainment, without MVPA guideline attainment = 0, meeting MVPA guideline attainment = 1

N = 188

Regarding depression, we found that daytime dysfunction (*β* = 0.319, *p* < 0.001) and MVPA guideline attainment (*β* = -0.302, *p* = 0.005) made a significant contribution and accounted for 25.7% of the variance of the depressive symptoms. Regarding anxiety, we found that BMI (*β* = 0.150, *p* = 0.041), sleep disturbance (*β* = 0.162, *p* = 0.032), daytime dysfunction (*β* = 0.402, *p* < 0.001), and MVPA guideline attainment (*β* = -0.218, *p* = 0.038) significantly contributed to the model. Regarding stress, BMI (*β* = 0.185, *p* = 0.017) and daytime dysfunction (*β* = 0.325, *p* < 0.001) significantly contributed to predicting the stress symptoms.

## Discussion

This study aimed to compare the differences in internalizing symptoms and physical activity between children with ADHD and sleep problems and children with ADHD but without sleep problems and to determine the factors associated with internalizing symptoms in children with ADHD.

The most important and novel finding was that 59% of children with ADHD were found to experience sleep problems. This result is in line with previous findings reporting the high prevalence rates of sleep problems ranging from 25 to 71% (Li et al., 2022; Sung et al., 2008) in children with ADHD. As expected, we found significant differences in internalizing symptoms and PA in children with ADHD and sleep problems and children with ADHD but without sleep problems. Consistent with previous studies, children with ADHD with moderate to severe sleep problems had more opportunities to experience both internalizing and externalizing comorbidities compared with those with no/mild sleep problems (Lycett et al., 2015); children with mild and moderate or severe sleep problems had lower quality of life scores and psychological scores than those without sleep problems (Sung et al., 2008); and children with ADHD and insomnia symptoms showed poorer sustained attention and visual-motor processing performance than children with ADHD without insomnia (Li et al., 2022). Thus, our results strengthened the point that children with ADHD and sleep disturbances had more severe internalizing symptoms than those without sleep problems. Additionally, our results showed that compared to children with ADHD but without sleep problems, children with ADHD and sleep disturbances engaged less time in MVPA daily (78.11 min/day Vs 66.70 min/day) and had a lower proportion to meet the MVPA guidelines (72.7% Vs 55.9%). Consistent with previous studies, youth with insufficient sleep duration (≤ 9 h/night) had significantly lower daily MVPA than youth sleeping longer than 10 h (Ortega et al., 2011). These novel findings strengthen the view that poor sleep may decrease children’s PA levels (Tambalis et al., 2018). Therefore, our study suggests that children with ADHD and co-occurring sleep problems present with a more severe clinical presentation of internalizing symptoms and physical inactivity. Previous studies suggested that both ADHD and sleep difficulties reflect a common physiological mechanism (e.g., irregular arousal) (Ball et al., 1997), causing them to be particularly vulnerable in specific domains (Bar et al., 2016), such as executive dysfunction, co-occurring psychiatric problems, and academic underachievement (Becker, 2020). Given the high comorbidity rates between ADHD and sleep, further studies should examine the interplay of sleep and other health behaviours among individuals with ADHD (Becker, 2020).

In the current study, MVPA guideline attainment negatively affects depressive and anxiety symptoms in children with ADHD. In line with a recent study, MVPA was negatively associated with depression and stress in children with ADHD (Liang et al., 2023b). Moreover, adolescents with ADHD who had higher-than-usual PA showed less depressed affect in the evening compared with those with lower-than-usual PA (Gawrilow et al., 2016), and better scores in health-related QoL were correlated with increased frequency of PA in children with ADHD (Gallego-Méndez et al., 2020). Consistent with children without disabilities, previous studies also reported that increased PA in childhood is related to a lower incidence of internalizing symptoms in adolescence and young adulthood (Wu et al., 2018) and higher MVPA at baseline was associated with lower symptoms of both depression and anxiety at follow-up (Buchan et al., 2021). In contrast, previous studies reported no prospective association between MVPA guideline adherence and depressive symptoms in youth (Patte et al., 2020) and that meeting the MVPA guideline was not related to flourishing, school engagement and BMI in children with ADHD (W. Wang et al., 2022). It is worth noting that previous findings were based on the proxy report, which may be prone to reporting bias. The current study utilized actigraph to objectively measure PA levels, confirming the associations between objectively assessed MVPA and mental health benefits in children with ADHD. In addition, we found that BMI was a significant predictor for developing anxiety and stress symptoms in children with ADHD. Consistent with previous studies, internalizing symptoms (e.g., distress and anxiety) are associated with increases in BMI in children (Ames et al., 2015; Gibson-Smith et al., 2020), and BMI and internalizing symptoms were reciprocally associated with each other for children 7–11 years old (Zhou et al., 2022). In children with ADHD specifically, there have been significant associations between ADHD and obesity/overweight (Cortese et al., 2016). The possible explanation is that children with ADHD and higher BMI are more likely to experience sleep disturbances, bullying and social exclusion and have low PA levels and a disrupted diet; thus, they may have a higher risk for the development of internalizing symptoms (Zhou et al., 2022).

The key finding of this study was that sleep-related daytime dysfunction (as measured by PSQI) was significantly associated with internalizing symptoms (i.e., depression, anxiety, and stress) in children with ADHD. Consistent with previous studies, sleep problems in children with ADHD predicted internalizing symptoms (i.e., depression) one year later after controlling for baseline symptoms (Becker et al., 2015); sleep problems at baseline predicted internalizing symptoms (i.e., emotional problems) at 6 months (Mulraney et al., 2016); and daytime sleepiness in children with anxiety disorders predicted internalizing problems (Hansen et al., 2014). Recent evidence has suggested that sleep problems may contribute to the development of internalizing and externalizing problems in children with ADHD and the relationship between sleep and internalizing and externalizing problems is bidirectional (Becker et al., 2015; Mulraney et al., 2016).It is possible that specific sleep problems, such as sleep disturbances or night awaking, may cause daytime sleepiness at school, and emotional and behavioural problems and poor academic performance at school may be criticised by teachers or parents, causing internalizing problems in children with ADHD over time (Lucas et al., 2019).

Our findings were well-aligned with those of previous studies showing that poor sleep quality, sleep latency, sleep disturbance and daytime dysfunction are positively correlated with internalizing symptoms (depression, anxiety, and stress) in children with ADHD (Lycett et al., 2015; Mayes et al., 2009). The novel findings of this study showed that meeting the WHO-recommended 60 min of MVPA guideline was negatively related to anxiety and stress in children with ADHD. In line with previous studies among the general population, children with ADHD who accumulated high PA levels experienced fewer mental health problems than those who had medium- and low-level PA (Ganjeh et al., 2021). As such, it is recommended that children with ADHD should do at least an average of 60 min per day of MVPA to improve impaired cognition, physical function, and mental health (World Health Organization, 2020).

This study is one of the very few that explored the effects of sleep on internalizing symptoms and physical activity in children with ADHD. A primary strength of this study was the use of actigraphy to objectively measure physical activity levels, which were not fully considered or adopted in previous research. Nonetheless, this study has some limitations. First, we used a cross-sectional design, which limits the possibility of making causal conclusions about sleep problems, internalizing symptoms, and PA. There is a need for longitudinal research to examine the causal mechanisms supporting our observations. Second, as the current findings on sleep quality and internalizing symptoms are based on child reports, our results may be prone to reporting biases. Further studies should be designed to use objective and reliable measures of sleep (e.g., polysomnography) and internalizing disorders biomarkers (e.g., cortisol) to gain a more nuanced understanding of sleep's effect on health-related outcomes in children with ADHD. Last, healthy controls were not recruited in the current study to have a comparison with the ADHD groups.

## Conclusion

The current study provides evidence that children with ADHD and sleep problems are less active and present with more severe internalizing symptoms than ADHD children without sleep problems. BMI, daytime dysfunction and MVPA guideline attainment predict the internalizing symptoms. Given the high rates of children with ADHD who experience sleep problems and children with ADHD without sleep problems spent more time in MVPA. Future research should consider the implementation of PA intervention, which may lead to improvements in sleep problems and internalizing symptoms in this clinical group.
